# Ovarian Dermoid Tumors

**Published:** 1876-04

**Authors:** W. H. Byford

**Affiliations:** Prof. of Obstetrics, etc., Chicago Medical College; Chicago


### OVARIAN DERMOID TUMORS.

Read to the Society of Physicians and Surgeons of Chicago, January 23, 1876,

By W. H. BYFORD, A.M., M.D.,

Prof. of Obstetrics, etc., Chicago Medical College.

Mr. President and Gentlemen:

In presenting this paper to you, I do so with special reference to what I consider a very remarkable case, which recently came under my observation. I therefore begin it with a history of that case.

Mrs. M., twenty-five years of age, was married in January, 1873. She has been delicate from childhood, and since the establishment of menstruation—although this function has been regular as to time and quantity—she has always suffered from pain at the periods.

During the summer of 1872, she had more pain at these times than usual, and in the intervals complained of backache and bearing-down sensations, to such a degree as to make it difficult to walk or be on her feet for any length of time. In October of the same year, she was so prostrated as to make it necessary to take her bed. In this condition, at the close of a menstrual flow, she was suddenly seized with greatly increased pain in the pelvis, heaviness, and difficult menstruation. This was followed for a number of days by moderate metrorhagia. The acuteness of the symptoms slowly subsided, but left her in a state of great prostration and suffering.

As soon as she was able to be placed in a palace car, she was brought to Chicago, accompanied by her physician, Dr. A. G. Jones, of Lexington, Ill. This was in May, 1873. She was then unable to walk more than a few steps at a time on account of debility and a severe bearing-down sensation.

Upon making a pelvic examination, I found a retro-uterine tumor filling up the *cul de sac* of Douglas. She complained of great tenderness when the uterus and surrounding parts were touched. The tumor was large enough to press the uterus up near the pubis, and fix it in that position. Any attempt to remove the uterus gave her great pain. The tumor could be indistinctly felt at the pelvic brim.

My diagnosis at the time was retro-uterine hematocele in a state of inflammation, and I expressed the opinion that it would probably suppurate. The pulse was accelerated, face flushed, tongue dry, and appetite poor.

I advised her to observe strict quiet in the recumbent posture, prescribed a supporting treatment and regimen, with occasional anodynes as the pain might render them necessary.

She went home, and returned to the city the following August, very much improved in general health. Although she was led to expect and watch for suppuration, she was not aware that there had been any purulent discharge. An examination at this time revealed the facts that the tumor was somewhat larger than when last seen, decidedly elastic, and more globular in form. As a diagnostic measure, with the assistance of Dr. Merriman, I introduced an exploring trochar and evacuated three or four ounces of clear, yellow serum. As the tumor collapsed and partially disappeared, I hoped this operation would prove curative in its results.

The patient left the city, but returned again in July, 1874, eleven months after the tapping. The tumor had commenced to fill again, but I thought it best to wait for further development, and sent her home.

I saw the patient again in April, 1875, and decided that the tumor was growing, as it could now be felt in both iliac and the hypogastric regions. I advised her this time to return to the city in the autumn, that I might operate for the evacuation and obliteration of the tumor.

She came to the city the 15th of October, 1875, and I found the tumor had grown quite decidedly since last examination. I determined to evacuate the tumor, and establish permanent drainage, with the hope of obliterating its cavity. Accordingly, assisted again by my colleague, Dr. H. P. Merriman, I operated by passing a large, long curved trochar into the tumor behind the uterus. Upon withdrawing the stylet, there flowed through the tube a thick, opaque, pearly-looking fluid, that reminded me of the tenacious albuminoid substance often seen to flow from multilocular ovarian cysts. From twelve to sixteen ounces were thus evacuated. The long canula was left *in situ*, and the patient placed in bed.

The tumor was very much diminished in size, but did not wholly disappear. The same kind of substance continued to flow for four or five days in small quantities. The cavity was washed out through the canula with a solution of chloride of sodium twice a day.

On the fifth day I removed the trochar, and placed in the perforation a large gum elastic catheter, and the cleansing injections were continued through it. Internally quinine, iron and the mineral acids were administered.

The tumor continued to diminish in size, but a constant discharge kept up, although steadily decreasing in quantity. The discharge at first was a greasy serum, with a peculiar odor. This became purulent, but at no time was there fetor of a character to indicate chemical decomposition, and before the death of the patient the quantity was very small in amount.

I could not detect the tumor above the pelvis, and by bimanual examination convinced myself that it was slowly disappearing.

The strength of the patient was well sustained up to the 1st of December, when she was attacked by slight chilliness, with a moderate acceleration of the pulse. At no time from the date of the operation had the pulse ranged higher than ninety beats to the minute. After the chills it mounted to 105. Tonic treatment, with nutritious diet, was kept up during the whole time.

The second day after the chills occurred, she was seized with diarrhœa. The stools were copious, frequent, watery, and very fetid. Unfortunately vomiting soon supervened, and she was unable to retain food or medicine in sufficient quantities to be of service to her. Great prostration and exhaustion rapidly succeeded, and in spite of every effort to sustain her, she died at midnight, December 25, 1875.

The *post-mortem* examination kindly made for me by Dr. W. H. Warn, revealed a small dermoid cyst that would have held six ounces of fluid—involving the left ovary—containing a roll of fine hair about the size of a pullet's egg. The hair was blond, although that of the patient was very black. It was matted together in a pretty compact mass, and formed of individual hairs from four to eight inches long.

This tuft of matted hair was saturated with the same tallow-like substance that had been drawn from the tumor when punctured, and with heat it could be melted and caused to run from it. The hair was lying loose in the cavity of the tumor, not the slightest attachment could be discovered anywhere. The lining membrane of the tumor presented a uniformly rugous dermoid appearance, and in places was in a state of suppurative inflammation.

At the time of the operation, about eight ounces of fluid was collected in a small earthenware bowl for examination. After standing a few moments, and becoming cool, it was found to be solid, and had assumed a buff color, having very much the appearance of mutton suet.

Upon closer examination, it was found to be oleaginous in all its properties. It melted on the fingers, and could not be washed off without soap. Heat melted it into an amber-colored oily fluid that was easily poured into a bottle, where it again solidified, and looked like a poor article of olive oil that had been submitted to a very low temperature. It burned through a wick like any other oil, and made a pleasant, bright yellow flame.

I sent a specimen of this substance to Dr. W. L. Atlee, of Philadelphia, who was kind enough to submit it to Dr. Drysdale for examination. Dr. D. says:

"The specimen was of a pale yellow color and odorless. It became liquid at a temperature of 84° Fahrenheit, and remained so at 82°, but commenced to solidify at 80°. Ether dissolves it readily and entirely, except some small flakes of a whitish color. It did not crystallize on cooling or evaporation of the ether.

"*Microscopic Appearances.*—The only distinct cell present was the scaly epithelial. It contains a large number of cells of this character, and remains of these cells partially disintegrated. A few cells of large size were also seen, and a great number of small, obscurely granulated cells, resembling the remains of pus cells. Except these cells, all were indistinct."

Dermoid cysts are not peculiar to the ovaries. They occur in various parts of the body, but in the ovaries they grow much larger than elsewhere, and the characteristic contents exist in greater quantities.

The cystic walls of the ovarian dermoid tumor are thicker and more dense than in the other varieties of ovarian cysts, and the lining membrane in some of its extent has a modified cutaneous structure—whence the name dermoid.

In tumors of this kind are found sebaceous or fatty matter, hair, bone, teeth, and, though rarely, muscular and nervous tissues. But these different substances are not often all found in every single specimen of this variety of tumor. Generally some one or two of them prevail to the exclusion of the others, as, for instance, bones, teeth and short hair, while in others the sebaceous material and long hair are associated without the bone.

In April last, I removed a tumor from a young Jewess which contained about twenty pounds of thin serum, bone, teeth, and short hair. The bone constituted a reticulated mass, two inches thick, and about eight inches in diameter, attached to the inside of the cyst. It somewhat resembled honeycomb in its arrangement, with interstitial cavities irregularly permeating it in every direction. To the surface of this bony accumulation were everywhere attached a multitude of rudimentary teeth, in appearance and structure precisely resembling the crown and body of the first incisors of a very young child. The enamel was complete, and the dentine beneath, quite perfect in structure. They were attached by a fibro-cellular substance, and could be easily separated by the fingers. I did not count them, but I am sure there were several scores. Among these dentoid bodies was quite a thick growth of short, very fine blond hair, from half an inch to two inches in length. The bone and teeth presented all the microscopic characters of these substances found in their normal relations.

Another ovarian dermoid tumor, removed from a young, healthy girl, at the Mercy Hospital, contained no bone, fat or teeth, but on one side the wall of the cyst was half an inch thick over a space as large as the palm of the hand. On the surface of this thickened portion there grew a tuft of very fine blond hair. Some of the hair was three inches long, but most of it from half an inch to an inch in length. The thickness of this part of the sac was caused by a mass of cartilage arranged in the same reticulated manner as the bone found in the case of the Jewess. There were from fifteen to twenty pounds of serum in this tumor, in which was discovered the granular cell of ovarian fluid.

The cyst, to which your attention is called to-night, yielded, at the first tapping in August, 1874, nothing but two or three ounces of serum, while on the 15th of October, 1875, there was evacuated nothing but a large quantity of sebaceous matter, of which this now submitted for your inspection is a sample.

At the autopsy we found the sac empty of everything but the bunch of hair here exhibited. There is no trace of bone, teeth, muscular or nerve tissue.

After making such research as my sources of information afford, I can find no instance of this kind of tumor in which this fatty matter preponderated. Usually it constitutes but a small part of the contents, the main bulk being serum.

In these specimens, we have something of the manner in which the different substances contained in dermoid tumors are distributed and associated.

The accepted theory of the formation of these tumors may be thus expressed : During early embryonic development a portion of the external layer of the blastodermic membrane, by some accident to the ovum, becomes depressed from the general level of the surface. The surrounding edges of the depression approximate over the depressed surface, come in contact, grow together, and thus enclose a dermoid cavity, in which the abnormal dermic products are generated. As the ovum grows, the adventitious cavity increases in size, and participating in the general genetic influence exerting itself in the whole embryo, its inner surface produces the rudimentary organs and secretions found in it.

When it is remembered that the external layer of the blastodermic membrane is the matrix in which are generated the skin and its appendages, and how extensively it engages in the formation of all the tissues and organs outside the abdomen, this theory will appear quite plausible.

The locality of the accidental blastodermic indentation must determine whether the dermoid inclusion will be in the dura-mater, scrotum, testicle, back of the neck, or ovary.

According to this theory, as well as established facts, this form of tumor is congenital.

In most localities it fails to attain great dimensions. When it is situated in the ovary, however, its growth is influenced by the great change which comes over that body at puberty, and that continues during the sexual activity of woman. As a consequence of the superior vitality of the ovary during that time, they are excited into rapid growth.

